# Immunogenicity assessment for vidutolimod: a risk-driven approach for a simplified 1-tiered, singlicate anti-drug antibody testing strategy

**DOI:** 10.3389/fimmu.2026.1751717

**Published:** 2026-05-19

**Authors:** Jenny L. Valentine, Stacey Shank, Lisette M. Fred Lucena, Zhiqiang Wang, Colleen McLaughlin Stern, Byung Chul Kim, Kun Lu, Saileta Prabhu, Michael A. Partridge

**Affiliations:** 1 Regeneron Pharmaceuticals, Inc., Tarrytown, NY, United States; 2 Immunologix Laboratories, Tampa, FL, United States

**Keywords:** 1-tier, anti-drug antibodies, bioanalytical strategy, immunogenicity risk assessment, singlicate

## Abstract

The bioanalytical strategy for a therapeutic should be driven by the overall immunogenicity risk of the molecule. Vidutolimod, a virus-like particle therapeutic, is being developed with a unique mechanism of action that requires the generation of a robust anti-drug antibody response to facilitate internalization and drive toll-like receptor immune activation. Consequently, the standard 3-tiered testing paradigm designed to detect very low levels of antibody may not be the best approach to generate clinically relevant data. The immunogenicity risk assessment performed for this virus-like particle led to the development of a simplified 1-tiered immunogenicity monitoring strategy where samples were tested in the titer assay in singlicate to determine both anti-drug antibody status and magnitude. This virus-like particle–based drug is an example of an alternative therapeutic modality where a thorough immunogenicity risk assessment was used to customize an immunogenicity testing strategy to meet the needs of the clinical program.

## 1 Introduction

For protein therapeutics, a major focus of immunogenicity assessment has been the ability to detect very low levels of anti-drug antibodies (ADAs) to ensure patient safety. As a result, regulatory guidance includes a 3-tiered analysis of samples, with screening, confirmation, and titer steps (1, 2). This paradigm was initially established in the context of high-risk biologics that mimicked endogenous biological counterparts where detection of ADAs was critical to understanding serious safety events (3). However, as we have built more knowledge about existing therapeutic modalities and while the drug landscape continues to expand to newer types of modalities, the utility of the very conservative 3-tiered testing paradigm has been repeatedly questioned (4–6).

ADA generation and its consequences can vary widely across therapeutics. As a result, in recent years health authorities and industry have recommended performing an immunogenicity risk assessment (IRA) to tailor the testing strategy based on the risk level and context of use of the therapy (1, 2, 7–9). Unwanted immunogenicity against a therapeutic through the generation of ADAs can impact pharmacokinetics, safety, and efficacy (2, 8). While sponsors need to understand the impact of ADAs on their programs, the level of characterization should reflect the risk of the particular drug candidate. An IRA is designed to assess product-, patient-, and study-related factors that impact ADA formation and its consequences, including patient population, product origin and structure, aggregates, and dose and frequency of administration. The information described in an IRA can help establish a bioanalytical sampling and testing strategy aligned with the treatment context and risk profile of a given therapeutic.

Similar to the 3-tiered testing paradigm, ADA sampling recommendations were also based on a particular context of use, in this case, traditional protein therapeutics such as monoclonal antibodies (mAbs). Within guidance and accepted literature (1, 10), there are recommendations to collect at early timepoints after initial dose, and intervals with increasing elapsed time for at least the first year, with minimal sampling thereafter. Using the information collected within an IRA, a sponsor can justify a sample collection strategy to obtain necessary immunogenicity information while minimizing patient burden.

This paper describes the immunogenicity assessment strategy for vidutolimod, a toll-like receptor 9 (TLR9) agonist. Vidutolimod consists of a cytidine phosphate guanosine type-A oligodeoxynucleotide that is encapsulated by a virus-like particle (VLP) composed of purified recombinant Qbeta (Qβ) bacteriophage capsid proteins. The VLP stimulates the production of anti-VLP antibodies, allowing vidutolimod to be taken up by plasmacytoid dendritic cells (pDCs). Once inside the pDC, the oligo is released to stimulate TLR9 and lead to the secretion of interferon alpha and other cytokines. Vidutolimod has been used in oncology trials in participants with diseases including melanoma, non-small cell lung cancer, and cutaneous squamous cell carcinoma, in addition to earlier studies in allergy indications (11–15).

Since vidutolimod was known to generate a robust ADA response that is required for the mechanism of action (MOA), a simplified testing strategy was designed and implemented for a phase 2 study in an oncology population. The strategy included development of a titer-based bridging immunogenicity assay to detect binding ADAs that was fully validated for 1-tiered, singlicate testing approach. This streamlined assessment approach balanced achieving a good sensitivity for the method (<100 ng/mL) while minimizing unnecessary analytical steps that historically have been used for protein therapeutics where detection of very low-level responses has been prioritized. Based on the risk assessment, testing for neutralizing ADAs was not performed.

## 2 Materials and methods

### 2.1 CMP-001–010 study information

CMP-001–010 was a multicenter, open-label, phase 2 study of intratumoral vidutolimod in combination with intravenous nivolumab in subjects with refractory unresectable or metastatic melanoma (NCT04698187). Participants received 10 mg of vidutolimod, given weekly for 7 weeks followed by every-3-week dosing. The first dose of CMP-001–10 mg was administered subcutaneously or by intratumoral injection, at the discretion of the investigator; all subsequent doses were planned to be administered intratumorally. Nivolumab was administered on day 1 of week 1 and every 3 weeks thereafter until progression, unacceptable toxicity, withdrawal of consent, or the end of study, whichever occurred first. A total of 44 participants were enrolled in the study. Blood samples for ADA analysis were collected at baseline, predose at week 7, at end of study, and every 3 months following completion of the treatment period. Blood samples for analysis of drug concentrations in serum were not collected in this study.

Informed consent was obtained from all individual participants included in the study and the study was conducted following all relevant ethical considerations.

### 2.2 Immunogenicity risk assessment

An immunogenicity risk assessment was performed using the principles outlined in health authority guidelines and industry white papers (2, 7, 8, 16). Briefly, potential risk factors related to product, participant, and trial design were considered. These potential risk factors included items such as drug product, CMC information, target biology, proposed indication and patient population, potential for previous exposure to similar products, pre-existing immunity, clinical study design, and concomitant medications. Any potential risk factors that could lead to an increase in the incidence of immunogenicity or consequences of immunogenicity were carefully considered to assign an overall risk level.

### 2.3 Vidutolimod ADA assay

The vidutolimod ADA assay was a semiquantitative, titer-based electrochemiluminescence stepwise bridging immunoassay run in singlicate. Vidutolimod was labeled with biotin at a 20:1 challenge ratio using EZ-Link™ Sulfo-NHS-LC-LC-Biotin (ThermoFisher Scientific, Waltham, MA, USA), then purified by Zeba™ desalting column (40 kDa MWCO) and stored in 1X Dulbecco’s phosphate-buffered saline (DPBS) with 0.01% Tween 20, pH 7.2, and with ruthenium N-hydroxysuccinimide (NHS) ester (Meso Scale Discovery [MSD], Gaithersburg, MD, USA) at a 20:1 challenge ratio, then purified with Zeba desalting column (40 kDa MWCO), also stored in 1X DPBS with 0.01% Tween 20, pH 7.2.

In the assay, the capture reagent, biotin-vidutolimod (2 µg/mL), was added to a blocked streptavidin-coated MSD microplate. Following an incubation step and 3X wash step, diluted samples and controls were added and allowed to incubate. Lastly, following an incubation step and 3X wash step, the detection reagent, ruthenium-vidutolimod (2 µg/mL), was added and allowed to incubate on the plate. A bridge formed when ADAs present in a sample bound to both labeled drug reagents. After a 3X wash step and addition of read buffer, the complexes were detected by electrochemiluminescence signal counts measured on a Sector Imager 600 plate reader (MSD), which were proportional to the amount of positive control or anti-vidutolimod antibodies in serum samples. The positive control was a humanized anti-Qβ immunoglobulin G1 subclass antibody, generated using *Escherichia* phage Qβ as the antigen (17).

The assay was developed and validated using principles outlined in health authority guidance documents and industry white papers (1, 2, 18, 19). For validation, a statistically determined cut point established with commercial samples obtained from individuals with solid tumors was set at a 1% false-positive rate (FPR). This cut point was used to determine positive responses in the validation. Due to high baseline positivity in study samples, an in-study cut point was used to determine positive responses during sample analysis. Samples were serially diluted (titered) using 5-fold dilutions for a total of 8 dilutions; responses greater than the cut point were considered positive for ADAs. The magnitude of the response was determined by end point titer, where the reported titer was the inverse of the highest dilution with a response in the assay greater than the cut point, or the highest dilution tested if the sample had a signal greater than the cut point at all dilutions.

### 2.4 Analysis of ADA data

Immunogenicity analyses were summarized descriptively, and no formal statistical comparisons were performed. The ADA analysis set (n = 41) included all participants who received any study drug and had ≥1 post-baseline ADA result. The ADA status of each participant was classified as negative, preexisting immunoreactivity, treatment-emergent response, or other positive response. Negative status was defined as all samples negative in the ADA assay, preexisting immunoreactivity was defined as a positive ADA result at baseline and all post-baseline results negative, treatment-emergent response was defined as a negative or missing baseline result with ≥1 positive result after the first dose, and treatment-boosted response was defined as a positive baseline result with ≥1 positive post-baseline result that was at least 25-fold higher than the baseline response. The relationship between ADA status and disease response was explored descriptively. Disease status was determined by the investigator and categorized as complete response, partial response, stable disease, progressive disease, or not evaluable. The relationship between maximum ADA titer and specific adverse events (AEs) including cytokine release syndrome, injection site reactions, and allergic reactions was explored descriptively.

## 3 Results

### 3.1 Immunogenicity risk assessment

Exploratory ADA analysis was previously performed on clinical study samples using a nonvalidated assay, which showed robust ADA response after administration of vidutolimod. Upon transfer of the asset to Regeneron, we initiated an IRA and made the decision to develop and validate a new ADA assay to assess future clinical ADA samples. As the previous data was generated from a nonvalidated assay, no cross-study comparison was performed.

The IRA performed for vidutolimod was used to develop a risk-based strategy for the clinical immunogenicity assay and future study sampling. The Key considerations are shown in **Table 1**.

**Table 1:** Key risk factors of vidutolimod.

Potential risk factor	Comment
Product origin	Since Qβ is a bacteriophage capsid protein, there is a high probability of ADA formation.
Molecular structure	Comprised of repeated Qβ units, which results in a high probability of ADA formation.
Mechanism of action	Generation of ADAs is required to opsonize the drug by plasmacytoid dendritic cells and release its oligonucleotide cargo to the intracellular TLR9 target. Therefore, there is a high probability of a strong ADA reaction.
Dosing	Increased risk of adverse events due to repeated administration in the presence of robust ADA responses.
Target indication	Participants with solid tumors are often immunocompromised from multiple treatments, reducing the risk of an immunogenic response.

ADA, anti-drug antibody; Qβ, Q beta; TLR9, toll-like receptor 9.

#### 3.1.1 Product-related risk factors

For vidutolimod, there is a very high probability of ADA generation against the repeating Qβ structure. The MOA of vidutolimod relies on the generation of ADAs against the VLP to opsonize the drug into pDCs (20). To achieve this MOA, vidutolimod consists of repeating, foreign Qβ particles on the exposed outer surface. The Qβ serves the purpose of both stimulating antibody generation for opsonization and forming the VLP to protect the oligonucleotide cargo until intracellular delivery. The MOA is supported by *in vitro* and *in vivo* data that prove that anti-Qβ (anti-VLP) antibodies need to be present for immune cell stimulation and induction of the antitumor response (12, 20). There is a very low probability of ADA generation against the G10 oligonucleotide cargo since G10 is not expected to be present in circulation for any significant length of time based on data that oligodinucleotides with a phosphodiester backbone are rapidly cleaved by nucleases (21–23).

Other product-related factors may reduce the probability of ADA generation. The manufacturing process is monitored with in-process controls, with steps designed to remove encapsulated host cell–derived nucleic acids and proteins and other process-related impurities. Product quality attributes for vidutolimod are carefully controlled by standard operating procedures and measured by release assays to maintain safe levels of aggregates, host cell protein, and host DNA.

Despite these purification and manufacturing control steps, the presence of foreign Qβ particles that stimulate ADA formation drives many of the decisions related to the immunogenicity assessment strategy. For vidutolimod, the risk of immunogenicity is high based on product-related risk factors.

#### 3.1.2 Patient-related risk factors

The ongoing open clinical studies were in various oncology indications, where participants were often immunocompromised and therefore at a lower risk of developing a strong immune response. To our knowledge, no approved drugs contain Qβ, so the risk of participants having antibodies that cross-react with vidutolimod is low. Consistent with this, previously reported data from participants receiving vidutolimod did not show a high rate of preexisting reactivity. Vidutolimod is given on an ongoing basis and has been administered intratumorally and subcutaneously, with flat doses ranging from 1 to 10 mg (11–15). Overall, the patient-related risk factors suggest a low contribution to the overall immunogenicity risk.

#### 3.1.3 Consequences of immunogenicity

For vidutolimod, the generation of ADAs has been shown to be necessary for its MOA (12, 20); therefore, a strong humoral response is tied to efficacy of the drug. Although vidutolimod is a therapeutic containing protein components, ADAs are a desired effect of administration (similar to a vaccine) and a lack of ADAs may correlate with a lack of efficacy. Consequently, an assessment of ADA magnitude may be important to the interpretation of clinical impact. Since all participants should develop robust ADAs that could increase drug clearance, systemic drug levels are expected to decrease rapidly. Since internalization of the active component of the drug (the oligonucleotide) is important for efficacy, the impact of ADAs on pharmacokinetics may not be considered a significant risk. As with all therapeutics, there is the possibility that ADAs could result in safety events or treatment-related reactions. Based on existing clinical data, there was no correlation between ADA levels and patient safety.

#### 3.1.4 Immunogenicity sampling strategy

To monitor and interpret immunogenicity for vidutolimod, a risk-based sampling strategy was proposed. Similar to other clinical programs, ADA samples should be collected predose to minimize drug interference, including collection of a baseline sample to understand and characterize any preexisting reactivity. Based on immunogenicity risk and use of the data for this drug, it may be important to understand the generation of ADAs in the first 10 weeks. We will be able to learn from established collections if there is a need to adjust sampling in future studies. In the CMP-001–010 study, samples were collected at baseline, week 7, at end of treatment, and during follow-up.

### 3.2 Overall immunogenicity risk

Based on the potential risk factors described above and highlighted in **Table 1**, the overall immunogenicity risk of vidutolimod can be described as a high likelihood of developing ADAs with a low risk of safety concerns due to immunogenicity. Although ADAs are likely to increase clearance of vidutolimod from circulation, the dosing strategy has taken this into account. Additionally, based on previous clinical studies, no correlation has been observed between the generation of ADAs and patient safety, including hypersensitivity and injection site reactions. Based on the MOA, the generation of ADAs may be linked to drug efficacy, with more ADAs potentially leading to better efficacy. Therefore, the nomenclature of the “risk” of immunogenicity from the generation of ADAs is not applicable for vidutolimod with respect to how it is typically understood for protein therapeutics, as here the greater risk may be low ADA generation that leads to a lack of efficacy.

### 3.3 Immunogenicity testing strategy

As outlined in the IRA, vidutolimod is not a traditional biologic. Therefore, the immunogenicity testing strategy was designed based on its context of use. A robust, drug-specific immune response was expected in all participants after treatment, similar to that expected from a vaccine. While no guidelines exist that focus on antibody testing for vaccines, white papers focus on reproducible detection of the antibody response (18, 19). This contrasts with immunogenicity assays to support protein-based therapeutics, where highly sensitive assays with high drug tolerance are valued. The vidutolimod assay strategy integrated lessons from both vaccine and therapeutic assays. Inclusion of both screening and confirmation steps during analysis to ensure detection of low-level ADA responses was not critical. Instead, as with methods to assess vaccine responses, the assay needed to have a wide dynamic range capable of measuring high ADA levels.

**Figure 1:**
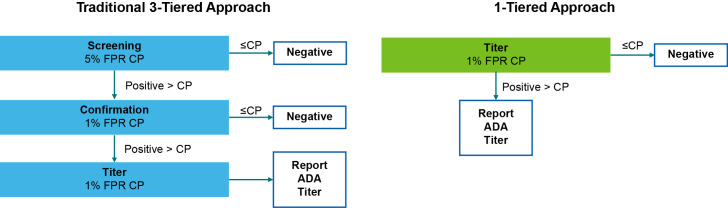
Classical 3-tiered testing strategy compared with the anti-vidutolimod antibody testing strategy. A 1-tiered approach was selected for anti-vidutolimod antibody testing. In blue, the traditional 3-tiered approach from guidance documents is shown, with samples first tested in screening using a 5% false-positive rate (FPR) cut point (CP). Samples that screen positive are then taken to the next tier of confirmation, which assesses samples using a 1% FPR CP. Any positive samples are finally analyzed in the titer tier using a 1% FPR CP. For vidutolimod, the 1-tiered approach is shown in green. All samples were analyzed in the titer tier using a 1% FPR CP. Samples where the initial dilution has a signal below the cut point in the titer assay are reported as below the MRD for the 3-tiered approach, while those same samples are reported as negative in the 1-tiered approach. For the 1-tiered approach, samples with signals above the cut point were reported as the inverse of the greatest titer value before crossing the CP or greater than the maximum titer if the signal was still above the CP at the eighth dilution. ADA, anti-drug antibody.

In addition, the robust immune response would result in a rapid decline of apparent drug levels in serum. For traditional protein therapeutics, this would be considered functional neutralization of the drug. For vidutolimod, this rapid uptake by immune cells is integral to the MOA and would, in contrast, be an indication of functional activity. Furthermore, the therapeutic was not an endogenous mimic, reducing the likelihood of serious clinical sequelae due to the formation of neutralizing responses. Therefore, a neutralizing antibody assay was not required to further characterize the ADA response. Taking all of this information into account, we planned a strategy to test study samples using a simplified, 1-tier approach using a statistically determined 1% FPR cut point, instead of the traditional 3-tiered approach used for standard protein therapeutics (**Figure 1**).

For this simplified analytical approach, we considered assay strategies employed for vaccines as there are similarities in the context of use since successful vaccines also expect a robust immune response (24). The most common approach for monitoring the magnitude of vaccine immunogenicity is to interpolate from a positive sample derived from a patient with efficacious response. In addition, signal-to-noise (S/N) and endpoint titer have also been proposed (18). Since there was no established correlation between anti-vidutolimod responses and efficacy of the therapy from a previous nonvalidated assay, the interpolation approach was not ideal. Endpoint titer was selected as an appropriate method to assess the magnitude of the ADA response, with the 1% FPR value used both to determine ADA status (positive/negative) and as the titer cut point.

#### 3.3.1 ADA assay development and reagent generation

An essential component of measuring the anti-vidutolimod antibody responses in study samples was developing and validating a method capable of detecting high levels of expected ADAs based on the MOA of vidutolimod. We selected an electrochemiluminescence assay format for its known wide dynamic range and extensive use for ADA assessments of protein therapeutics, knowing that successful implementation of this assay format would hinge on the ability to generate the drug conjugates. Due to the size and structure of the VLP, we were unsure what challenges biotin and ruthenium conjugation would present. Some modifications were made to the labeling process to ensure optimal incorporation of both biotin and ruthenium labels. Initially, higher labeling challenge ratios (e.g., 500:1) were used but this proved to be unnecessary when tested in bridging ADA method format; in fact, preliminary testing of these reagents generated poor assay signal with a strong hook effect, suggesting that the drug was over-labeled when higher challenge ratios were used. A final challenge ratio of 20:1, similar to that employed for mAbs, was utilized for both biotinylated- and ruthenylated-vidutolimod. Conjugation of the drug was shown not to interfere with its binding with a purified mAb specific against Qβ (not shown).

**Figure 2:**
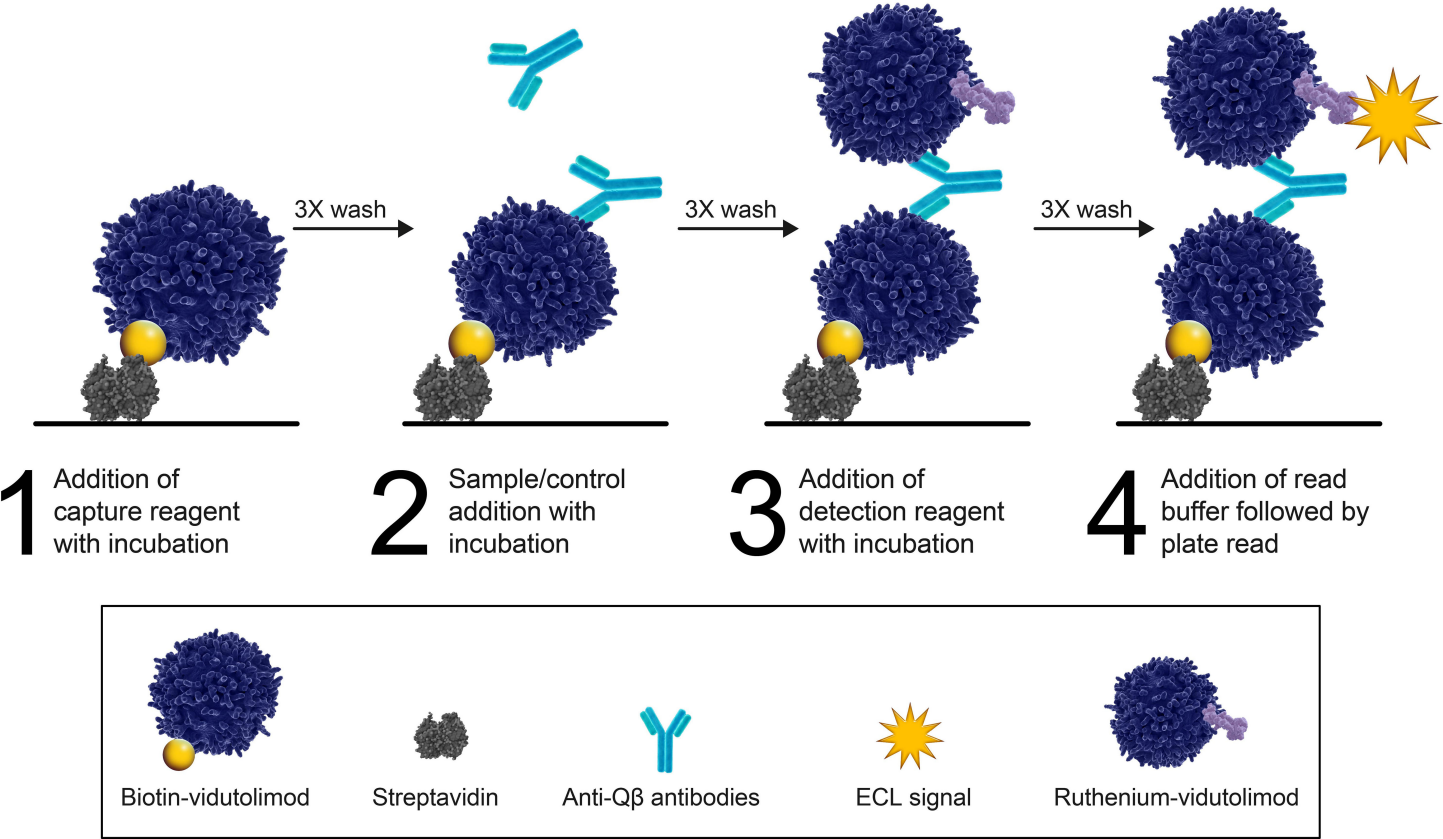
Stepwise bridging electrochemiluminescence assay format for the semi-quantitative measurement of anti-vidutolimod antibodies. Stepwise addition of biotinylated drug, samples, or controls, and finally ruthenylated drug allows for the positive control or anti-Qβ antibodies present in samples to bridge the labeled drug reagents.

Once conjugates were generated, they were tested in both homogenous and stepwise bridging assay formats. A hook effect/prozone was observed when using a homogeneous bridging format, which was mitigated and background signal decreased when a stepwise assay format was used. It was hypothesized that due to the high molecular weight of vidutolimod and relatively high density of Qβ epitopes on the VLP surface, both arms of the positive control antibody bound to a single vidutolimod molecule, resulting in a lack of bridge formation. By initially immobilizing the biotin-vidutolimod on the plate, followed by the addition of diluted sample containing ADAs and addition of the ruthenium-labeled vidutolimod, the accessibility of the Qβ for both arms of the positive control antibody was decreased and provided the optimal assay format (**Figure 2**).

#### 3.3.2 MRD and serial dilution scheme

Because the method required a large dynamic range and sensitivity was not a primary concern, a 1:250 minimum required dilution (MRD) was employed. Although this is greater than the 1:100 MRD recommended in health authority guidance documents (1), the sensitivity of the method was <100 ng/mL and therefore was appropriate for this context of use. In addition, a 5-fold serial dilution was used for sample analysis for a maximum of 8 dilutions to minimize sample handling and ensure capture of an appropriate titer result for all samples without repeated dilutions. By using this approach, the magnitude of responses detected would cover a large range of titers, spanning from 250 to ~1.9 x 10^7^. Samples below the cut point at MRD would be considered negative and samples that did not cross the cut point at the final dilution would be reported as greater than the highest titer (**Figure 1**). This approach would allow us to simplify titer testing and avoid fully titering extremely high magnitude ADAs that would be unlikely to be associated with differences in impact on efficacy or safety.

Since we would be diluting all samples for a total of 8 dilutions each, we tested singlicate vs duplicate analysis during method development. Singlicate analysis has been a topic of conversation in industry, with successful implementation in both pharmacokinetic and ADA ligand-binding antibody formats (25–29). In the case of the anti-vidutolimod ADA assay, the assay performance and results were consistent between singlicate and duplicate formats and had no impact to the overall conclusions. Therefore, to simplify the testing strategy further, we decided to utilize a singlicate testing for our 1-tiered titer assay.

#### 3.3.3 ADA assay validation

Based on the immunogenicity risk of vidutolimod, the requirements for validation of the stepwise bridging anti-vidutolimod ADA assay were aligned with the context of use of the assay. The acceptance criteria for various assay parameters are shown in **Table 2**, along with the results from the validation. Several assay parameters aimed to align with regulatory guidance for ADA assays, including assay sensitivity, inter-assay precision, selectivity, and stability. However, no specific acceptance criteria were established for parameters such as drug tolerance, since drug was given at low doses and expected to clear quickly from the bloodstream and therefore was not anticipated to interfere with the detection of robust ADA responses. Instead, those parameters were performed during validation and the results were reported as observed.

**Table 2:** Parameters of the validated anti-vidutolimod assay.

Assay parameter	Validation acceptance criteria	Anti-vidutolimod validation assay
MRD	No specified acceptance criteria (FDA guidance recommends ≤100)	250
Cut point factor	No specified acceptance criteria	1.166
Number of replicates	No specified acceptance criteria	Singlicate
Assay sensitivity	≤100 ng/mL	<50 ng/mL
Precision	%CV of PCs: ≤20%	Inter-assay: 9.7-19.1% Intra-assay: 3.0-10.2%
Selectivity (matrix effect) of disease-specific individuals	Unspiked samples: ≥80% negative LPC-spiked: 100% must be positive	Unspiked: 100% were negative LPC-spiked: 100% were positive
Selectivity of hemolyzed samples	No specified acceptance criteria	Unspiked: 100% were negative LPC-spiked: 100% were positive
Selectivity of lipemic samples	No specified acceptance criteria	Unspiked: 3/5 samples tested were negative LPC-spiked: 100% were positive
Drug tolerance	No specified acceptance criteria	HPC: >100 µg/mL drug MPC: 3 µg/mL drug LPC: <2 µg/mL drug
Interference of co-administered drugs*	No specified acceptance criteria	Method tolerates 1000 µg/mL of co-administered drugs
F/T, Bench-top (RT), and 2-8°C short-term stability of positive control	Two-thirds of stability samples ≥CP and rank appropriately in signal	F/T: 5 cycles Bench-Top (RT): >24 h 2-8°C: >24 h
Robustness (including different analysts, equipment/instrumentation, incubation times)	Acceptable validation runs summarized to establish incubation time ranges	Robustness was demonstrated and established upper and lower limit of incubation time ranges
Specificity with unrelated VLP	No specified acceptance criteria	LPC-specific >20 µg/mL unrelated VLP NC-specific >20 µg/mL unrelated VLP

*Vidutolimod DP is intended to be administered in combination therapy.

CP, cut point; CV, coefficient of variation; FDA, Food and Drug Administration; F/T, freeze-thaw; HPC, high positive control (5000 ng/mL); LPC, low positive control (100 ng/mL); MPC, medium positive control (1000 ng/mL); MRD, minimum required dilution; NC, negative control; PC, positive control; RT, room temperature; VLP, virus-like particle.

Drug tolerance is shown as the highest drug concentration for which PC is still detected.

### 3.4 Clinical ADA data from study CMP-001-010

The validated ADA assay was used to test clinical serum samples from study CMP-001-010. During sample analysis, a high baseline positivity was observed (>70%), which was significantly higher than the data obtained during method development and validation using commercial samples obtained from individuals with solid tumors, where significant preexisting ADA responses were not detected. An analysis of the distribution of the baseline responses indicated a higher average signal for the in-study samples, suggesting that the false positivity was due to population differences rather than preexisting reactivity in a subset of samples. Therefore, an in-study cut point of 1.653 was calculated using the existing baseline signal generated for all samples and, subsequently, all data were re-evaluated. The in-study cut point was set at a 1% FPR, similar to the validated cut point.

**Figure 3:**
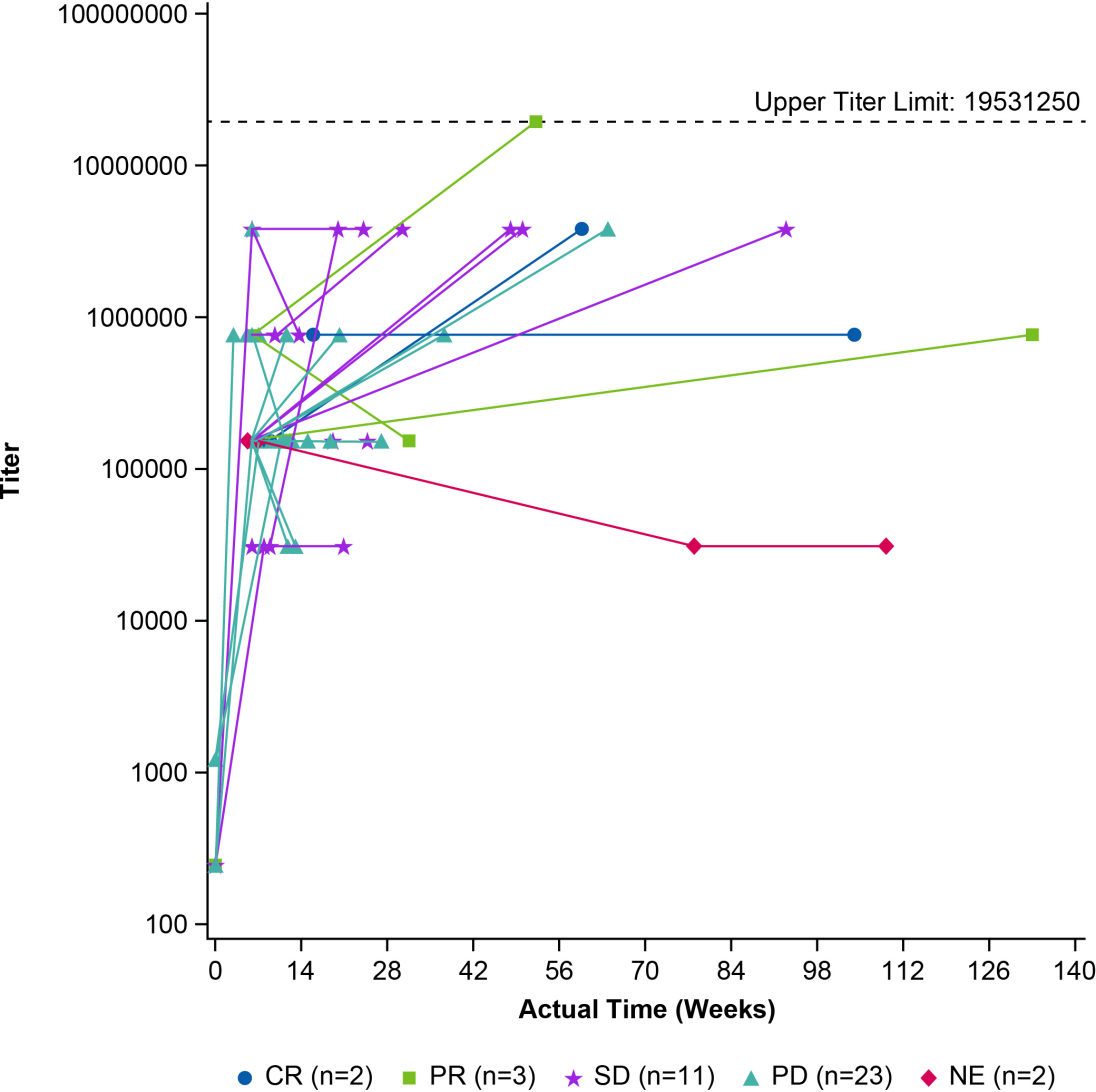
Longitudinal anti-drug antibody titers in participants with refractory unresectable or metastatic melanoma treated intratumorally with vidutolimod. ADA titers over time in participants enrolled in study CMP-001-010, an open-label phase 2 study, are shown. Subjects received vidutolimod at 10 mg every week for 7 weeks and then switched to an every-3-week dosing regimen. ADAs were measured using a validated assay in samples collected at baseline, pre-dose at week 7, at end of study, and every 3 months following completion of the treatment period. Each line represents an individual participant. Blue circle, light green square, purple star, teal triangle, and red diamond shapes indicate subjects who achieved a complete response (CR) (n = 2), partial response (PR) (n = 3), stable disease (SD) (n = 11), progressive disease (PD) (n = 23), and not evaluable (NE) (n = 2), respectively. ADA titers, presented in log scale, were reported as the inverse of the highest dilution with a response greater than the cut point, or the highest dilution tested if all dilutions had a signal greater than the cut point. The dashed black line represents the upper titer limit of 19,531,250. Titer value greater than upper limit tested (19,531,250) was set as the upper limit. n, number of participants.

The immunogenicity data from study CMP-001–010 using the in-study cut point are presented in **Table 3**. All participants (ADA analysis set, n = 41) showed a positive ADA response, as expected. The magnitude and kinetics of the titer response for each individual were color-coded based on disease status, as shown in **Figure 3**. By week 7, all participants had a positive ADA response. Similar to data generated from the previous clinical studies using the non-validated ADA assay (not shown), there was no correlation between either maximum ADA titer or generation of responses at protocol timepoints with the disease status classification for participants in the study.

**Table 3:** Summary of ADA status and ADA category by disease status classification in participants with refractory unresectable or metastatic melanoma (study CMP-001-010, AAS).

ADA status/category, n (%)	CR	PR	SD	PD	NE
ADA analysis set	2 (100)	3 (100)	11 (100)	23 (100)	2 (100)
Negative	0	0	0	0	0
Preexisting immunoreactivity	0	0	0	0	0
Treatment-emergent response	2 (100)	2 (66.7)	8 (72.7)	19 (82.6)	2 (100)
Treatment-boosted response	0	1 (33.3)	3 (27.3)	4 (17.4)	0

AAS, anti-drug antibody analysis set; ADA, anti-drug antibody; CR, complete response; n, number of participants; NE, not evaluable; PD, progressive disease; PR, partial response; SD, stable disease.

Percentages are based on ADA analysis set for ADA status. Negative ADA status indicates that all ADA samples tested negative. Preexisting immunoreactivity indicates that only the baseline sample tested positive. Treatment-emergent response indicates that the baseline sample was either negative or missing, and ≥1 sample tested positive after the first dose. Treatment-boosted response indicates that the baseline sample tested positive, and ≥1 additional sample tested positive after the first dose that was at least 25-fold higher than the baseline response.

An assessment of ADA response and AEs was also performed. Since all participants had high titers, the maximum titer responses for all participants were grouped into three sets and the percentage of participants with certain AEs of special interest was calculated (**Table 4**). Similar to previous data, there was no correlation between maximum ADA titer and AEs associated with the presence of ADA. The only grade-3 AE in categories associated with the presence of ADA was not associated with the highest maximum titer. In fact, the participant with the highest maximum titer did not have any AE identified in the categories assessed, further suggesting a lack of correlation between maximum ADA titer and AEs.

**Table 4:** Summary of maximum titer category by treatment-emergent adverse events in participants with refractory unresectable or metastatic melanoma (study CMP-001-010, AAS).

Maximum titer category	ADA analysis set, n	Participants with any-grade CRS, n (%)	Participants with any-grade ISR, n (%)	Participants with any–grade allergic reactions, n (%)
≤156,250	18	0	3 (16.7)	0
781,250	12	2 (16.7)	0	0
≥3,906,250	11	1 (9.1)	0	0

AAS, anti-drug antibody analysis set; ADA, anti-drug antibody; CRS, cytokine release syndrome; IRS, injection site reaction; n, number of participants.

Percentages are based on ADA analysis set. Allergic reactions include hypersensitivity and anaphylaxis. One participant with a maximum titer of 781,250 had a grade-3 CRS; all other adverse events were of grades 1 or 2.

## 4 Discussion

Assessing immunogenicity risk is an important step in establishing an immunogenicity monitoring and testing strategy that is appropriate for a particular therapeutic. A thorough IRA should both assess the likelihood of ADA generation and consider the clinical consequences should an immune response develop. By evaluating the risk of an immune response, appropriate monitoring and mitigation strategies can be put into place. For low-risk therapeutics, a risk-based approach for immunogenicity may mean modification of testing and collection strategy. This could include sparse sampling, omitting neutralizing antibody testing, or “collect and hold,” with ADA testing only performed under certain circumstances (eg, safety events) (30). High-risk therapeutics, characterized based on the risk of severe safety consequences due to ADAs in participants receiving the drug, may require more frequent sampling and early implementation of assays to monitor binding ADA and neutralizing antibody development.

An IRA for vidutolimod revealed a high probability of ADA generation against the outer structure coupled with a low likelihood of negative impact from its presence, overall resulting in a low safety risk of ADAs. Based on this risk level, we developed a 1-tiered assessment strategy wherein samples were tested only in singlicate in a titer assay against vidutolimod using a cut point with a 1% FPR. An ADA assay against the oligonucleotide cargo was not developed, as internal data revealed that vidutolimod is stable in serum. Therefore, the oligo would be unlikely to be in systemic circulation to generate an ADA response. Furthermore, even if a low amount was released extracellularly, nucleases in the blood would be expected to quickly degrade the unmodified oligonucleotide. This assay strategy is similar to the immunogenicity assessment approach for some vaccines since the method is designed to consistently detect high levels of ADAs across studies (18, 24). Since the MOA of vidutolimod involves the generation of ADAs, there was a possibility that ADA levels could correlate with clinical outcome. However, based on data from previous clinical studies generated from a nonvalidated method, it was unlikely that we would observe a correlation (not shown). A decision was made to increase the dilution steps during sample titration to make analysis more efficient and avoid excessive repeat titrations of high-magnitude ADAs. To that point, we considered an alternative approach to use a single tier of screening and report the S/N of each sample. Ultimately, challenges in reporting that data within the existing systems (and the lack of a known ADA level that would be associated with a clinical response) led to the decision to use the titer-based assay. An in-study cut point was calculated, as the validated cut point did not reflect the sample data from CMP-001-010, despite the use of appropriate disease matrix in setting the cut point during validation. It is worth noting that if S/N had been used to assess the ADA magnitude, it could have circumvented the need for validated and in-study cut points and simplified data analysis. As expected, study data obtained using this 1-tiered monitoring approach revealed that a strong ADA response developed in all participants, with no apparent correlation between the level of response and efficacy or safety outcomes.

Based on the context of use for the immunogenicity assay, we focused on certain assay specifications during development that were different from those of a traditional mAb therapeutic. For example, due to the robust immune response, we were less concerned with low levels of sensitivity and needed to ensure that the assay had a high dynamic range. Additionally, we did not expect drug tolerance to pose a significant challenge, as high levels of ADAs were expected to clear the drug quickly from systemic circulation. In this clinical study administering vidutolimod in refractory unresectable or metastatic melanoma, using the validated ADA assay did not reveal a correlation between ADA titer and disease status classification.

With the rise of newer modalities such as cell and gene therapies, oligonucleotide therapies and others, it has become clear that a risk-based approach is essential to developing an appropriate immunogenicity monitoring and testing strategy. Immunogenicity assays have expanded from traditional plate-based enzyme-linked immunosorbent assay and cell-based methods to a host of other technologies, including multiplex assays, flow cytometry, and mass spectrometry. Design of the appropriate assay strategy and choosing the appropriate platform for assay development can be informed by an IRA, which should describe the context of use for a given therapeutic. Additionally, the information contained in an IRA can be used in the Integrated Summary of Immunogenicity, a document that is recommended by multiple regulatory agencies to be included in submissions to provide a clear and concise summary of immunogenicity risk, mitigation, monitoring, and clinical data (1, 2).

Vidutolimod is an unusual therapeutic modality with a MOA that is very different from traditional protein therapeutics. Therefore, a wider range of potential ADA assessment approaches were considered, closely informed by the overall immunogenicity risk of the therapeutic. Ultimately, a nontraditional ADA testing approach was implemented that nevertheless provided meaningful data to support the clinical program.

## Data Availability

Qualified researchers may request access to study documents (including the study protocol with any amendments, statistical analysis plan) that support the methods and findings reported in this manuscript. Individual anonymized participant data will be considered for sharing 1) once the product and indication has been approved by major health authorities (e.g., FDA, EMA, PMDA, etc) or development of the product has been discontinued globally for all indications and there are no plans for future development 2) if there is legal authority to share the data and 3) there is not a reasonable likelihood of participant re-identification. Submit requests to https://vivli.org/.
