# Supplementary material for: Immunogenicity assessment for vidutolimod: a risk-driven approach for a simplified 1-tiered, singlicate anti-drug antibody testing strategy
Source: Front Immunol. 2026 May 19;17:1751717. doi: 10.3389/fimmu.2026.1751717 (PMC13227480; doi:10.3389/fimmu.2026.1751717)
Supplement: Supplementary file 1 [file Table1.doc]

**Supplementary Table 1. Institutional Review Board or Independent Ethics Committee List.**

| **Type of Center**  **(1 or 2)*** | **Study Center No.** | **Principal Investigator**  **Last Name & First Name** | **IRB/IEC Name and Address** | | **IRB/IEC Committee Chairperson**  **Last Name and First Name** | |
| --- | --- | --- | --- | --- | --- | --- |
|  |  |  | **Local IRB/IEC** | **Central IRB/IEC** | **Local IRB/IEC** | **Central IRB/IEC** |
| 1  2  1  2  1  1  1  1  1  2  1 | 103  117  121  125  135  136  138  142  145  146  148 | Kirkwood, John  Kendra, Kari  Bhatia, Shailender  Kudchadkar, Ragini  Hu-Lieskovan, Siwen  Westin, Gustavo  Naqash, Abdul Rafeh  Chandra, Sunandana  Thomas, Sajeve  McClay, Edward  Cowey, Charles |  | WCG IRB  212 Carnegie Center, Suite 301  Princeton, NJ 08540, USA |  | Fitzgerald, Kelly |
| 1 | 102 | Milhem, Mohammed | The University of Iowa Human Subjects Office IRB,  105 Hardin Library for the Health Sciences,  600 Newton Road,  Iowa City, Iowa 52242-1098, United States |  | Bertolatus, J. Andrew |  |
| 1 | 118 | Izar, Benjamin | Columbia University Institutional Review Board, Russell Hall, 154 Haven Avenue, Floor 2,  New York, NY, USA, 10032 |  | Lamanna, Nicole |  |
| 1 | 119 | Beasley, Georgia | Duke University Health System (DUHS) IRB, DUHS IRB, Suite 900 Erwin Square, 2200 West Main Street, Campus Box # 104026, Durham, NC 27705, United States |  | Diehl, Louis |  |
| 2 | 133 | Seetharam, Mahesh | Mayo Clinic Institutional Review Board, Mayo Clinic,  200 First St. SW,  Rochester, MN 55905, United States |  | Sher, Taimur |  |
| 1 | 143 | Shoushtari, Alexander | Memorial Sloan Kettering Cancer Center IRB/Privacy Board, 1275 York Avenue, New York, NY 10065, United States |  | Kaley, Thomas |  |

IEB, Institutional Review Board; IEC, Independent Ethics Committee
